# Comparison of the efficacy of Er,Cr:YSGG laser on oral biofilm removal from implant surfaces with various application times for the treatment of peri-implantitis defects: ex vivo study

**DOI:** 10.1186/s12903-024-04698-5

**Published:** 2024-08-22

**Authors:** Alaa Hashim, Nevine H. Kheir El Din, Nashwa El-Khazragy, Hadeel Gamal Almalahy

**Affiliations:** 1 Department of Oral Medicine, Periodontology and Oral Diagnosis, Faculty of Dentistry, Ain Shams University, Cairo, Egypt; 2 Department of Clinical Pathology-Hematology, Faculty of Medicine, Ain Shams University, Cairo, Egypt; 3 Ain Shams Medical Research Institute (MASRI), Faculty of Medicine, Ain Shams University, Cairo, Egypt

**Keywords:** Er, Er,Cr: YSGG laser, Peri-implantitis, 3D-printed model, Infrabony defects, Biofilm removal, Decontamination efficacy

## Abstract

**Purpose:**

The major struggle in peri-implantitis therapy is the availability of successful decontamination of the infected implant surface. The main hypothesis of this study was the Er,Cr: YSGG laser decontamination efficacy investigation on the infected implant surfaces with various peri-implantitis defects. The primary objective of this study was to decide the efficacy of Er,Cr:YSGG laser as a decontamination tool at various peri-implantitis simulating defects. The secondary objective was to compare the efficacy of the Er,Cr: YSGG laser on oral biofilm removal between two protocols the first protocol (4 cycles at 2.5 min) and the second protocol (5 cycles at 5 min) at various peri-implantitis simulating defects.

**Materials and methods:**

A total of 3 subjects whose plaque biofilms formed in-vivo on twenty-four tested implants were divided into four tested groups. Two native implants were tested as controls.The in vitro defect model was computer‐aided designed and printed into a 3D-printed model with various anulations in peri-implant infrabony defects, which were 15,30,60,and 90 degrees.

**Results:**

Both Er, Cr: YSGG decontamination protocols at 50 mJ (1.5 W/30 Hz), 50% air, and 40% water were effective at reducing the total implant surface area/ biofilm ratio (%), but the second protocol had a markedly greater reduction in the duration of application (5 cycles at 5 min) than did the first protocol (4 cycles at 2.5 min).

**Conclusion:**

The Er, Cr: YSGG laser is an effective decontamination device in various peri-implantitis defects. The second protocol(5 cycles at 5 min) with greater application time and circles is more effective than the first one. The defect angulation influence the decontamination capability in peri-implantitis therapy.

**Clinical relevance (Scientific rationale for study):**

Clinicians anticipate that the exploration of suitable therapeutic modalities for peri-implantitis therapy is limited by the obvious heterogeneity of the available evidence in the literature and need for a pre-clinical theoretical basis setup. The major challenges associated with peri-implantitis therapy include the successful decontamination of the infected implant surface, the absence of any damage to the treated implant surface with adequate surface roughness, and the biocompatibility of the implant surface, which allows osteoblastic cells to grow on the treated surface and is the key for successful re-osseointegration. Therefore, these are the expected empirical triads that need to be respected for successful peri-implantitis therapy. Failure of one of the triads represents a peri-implantitis therapeutic failure. The Er, Cr: YSGG laser is regarded as one of the expected devices for achieving the required triad.

**Trial registration:**

"Efficacy of Er,Cr YSGG Laser in Treatment of Peri-implantitis". ClinicalTrials.gov ID NCT05137821. First Posted date: 30 -11–2021.

**Supplementary Information:**

The online version contains supplementary material available at 10.1186/s12903-024-04698-5.

## Introduction

Currently, peri-implant diseases are regarded as one of the most critical issues with a high prevalence in various populations with progress in prosthetic therapies [1]. However, till the moment, there has been confusion regarding the exact pathological mechanism of peri-implantitis. It is considered a plaque-correlated multifactorial disease with subsequent progressive supporting bone loss. Quick intervention to block the progression of the disease or even tissue regeneration is essential [2]. The etiology, pathophysiology, and clinical manifestations of peri-implantitis are similar to those of periodontitis,; hence, periodontitis treatment nonsurgical or surgical approaches have also been applied to manage peri-implantitis [3].

Although the incidence of peri-implantitis is high, their primary management is still questioned with the absence of a single clear therapeutic protocol because there is insufficient evidence regarding specific practices [4]. The primary concept of peri-implantitis therapy is bacterial count eradication through biofilm disruption with preceding suppression of infection and inflammation, which are essential steps for stopping disease progression and re-osseointegration initiation [5]. The other concept is to have a new surface that achieves a proper osseointegration with the avoidance of bacterial colonization, and the reconstruction of lost tissues [6, 7]. Generally, long-term stability and re-osseointegration are typically seen as the purpose of peri-implantitis therapy [8].

One of the principal challenges in peri-implantitis therapy is the diverse implant body-related features and thread design, which impedes the accessibility of biofilm decontamination with various instruments. These challenges are evident in nonsurgical therapy circumstance, where the operator is "blind" to the biofilm regions [9, 10]. Moreover,the morphology of various peri-implantitis defects influences their healing potential in regenerative therapy [11, 12]. The progression of peri-implantitis defects is correlated with certain site specific, patient-related, and implant-related variables that are interrelated with defect morphology and severity [13].

The probability of re-osseointegration is evident around appropriately cleaned implants with direct structural and functional attachment between previously infected implants and bone [14]. The bacterial byproducts that persist on the implant surface are supposed to generate fibrous encapsulation, unlike on the pristine implant surface. In turn, a meticulous decontamination process is essential for re-osseointegration in peri-implantitis. In addition, the relationship between surface roughness and the osteoblastic response is proven [14, 15].

Accordingly, obtaining innovative and reliable approaches that evade undesirable alterations in surface topography or other biological side effects is critical. Unfortunately, there is no optimal strategy that has successful and predictable results until now [7, 16, 17].

Consequently, a continuous trial to retrieve other approaches to overcome the drawbacks of conventional therapy, such as Er, Cr: YSGG laser, or electrochemical therapy has been suggested with slight beneficial effects that need to be confirmed by long-term clinical studies with comparable groups [18, 19].

In the field of implantology, a suitable laser tool is proposed to have a reasonable efficacy for biofilm eradication, negligible absorption through the titanium body with a slight subsequent risk of titanium body temperature elevation, a negligible risk to surface morphology, acceptable soft tissue and hard tissue ablation, increased new tissue formation, and satisfactory antimicrobial value [20]. Therefore, it is valuable to balance the laser settings applied for debridement against any possible negative effects on the metallic implant surface and temperature rise [21]. The damage of the dental and periodontal tissues caused by laser-induced heat is evident with the wrong choice of laser wavelength or parameters that can cause morphological damage, including fractures or carbonization of the enamel surface, pulpitis, necrosis of the pulp, and injuries to the periodontal ligament or bone [22].

The choice of the safest laser therapy is guided by much more than just matching the emission spectrum of lasers to the absorption spectra of tissues. The correct wavelength selection is not the only decisive variable for therapeutic success. The appropriate laser parameters (pulse, duration of contact, peak laser power in hertz, and energy of emitting optic fiber in joules) and the application mode are more critical; this is precisely why laser training is crucial. Therefore, the quality of the target tissue must be judged distinctly in distinct cases of therapy at any wavelength, simply by modifying the laser parameters to outfit the target tissue with a suitable application mode [23].

Although there are insufficient data to determine the long-term benefits of laser therapy over conventional treatment, it is effective for treating peri-implantitis for up to three months [24]. Reconstructive treatment has suggested a favorable outcome for re-osseointegration with histologic signs of bone growth in human experiments following decontamination with the (Er, Cr: YSGG) laser. This outcome was measured by reestablishing bone-to-implant contact on contaminated dental implant surfaces. Clinic-based peri-implantitis treatment with the Er, Cr: YSGG laser is considered a viable option based on these human histologic results [25].

The main hypothesis of this study was the efficacy of Er,Cr:YSGG laser in decontamination of the infected implant surfaces with various peri-implantitis defects. This study was conducted in the form of a PICO Question (problem (peri-implantitis), Intervention (Er: YSGG implant surface decontamination in diverse bone defect simulators), Comparative (native implants), Outcome (oral biofilm removal)) [26]. The primary objective of this study was to evaluate the efficacy of Er,Cr:YSGG laser in oral biofilm removal from the implant surface at various peri-implant defect morphologies as a decontamination tool in peri-implantitis therapy. The secondary objective was to compare the efficacy of the Er,Cr: YSGG laser on oral biofilm removal between two different protocols, the first protocol (4 cycles at 2.5 min) and the second protocol (5 cycles at 5 min) at various peri-implantitis simulating defects.

## Subjects and methods

### Overall study design and volunteers selection

This study was designed and employed as an ex-vivo study with twenty-six (26) implants evaluated, including twenty-four (24) tested implants in the four groups and two (2) implants in the control group. Six implants from each group were allocated randomly by random number generation in Microsoft Excel. Two other new implants will be assessed as controls. The study protocol was reviewed and approved by the Research Ethics Committee Faculty of Dentistry;, Ain Shams University, with acceptance number (FDASU-Rec ID210314). The study was conducted at the laser center, of Ain Shams University. The protocol was registered in the U.S. National Institutes of Health Clinical Trials Registry (clinical trial.gov) at 30–11-2021, and the clinical trial ID number was NCT05137821. A preceding power analysis revealed that the necessary sample size was 8 implants (2 in each group) to detect a large effect size of 17.4 at an alpha of 0.05 and a power of 0.80 [27].

A specially designed 3D-printed model was created through computer‐aided design through specific CAD software (Exocad Dental CAD; Exocad), with four different infrabony defects that resembled human peri-implant defects created through a 3D printer (AccuafabD1s (Shining3D, China)) using specific resin material (Proshape digital solutions).This approach made it simple to change and test implant specimens in vitro [27]*.*

Peri-implant simulated defects were developed using Sahrmann's methodology. These circumferentially standardized, 6 mm-deep artificial infrabony defects differed only in their angulation which was intended to provide different levels of decontamination accessibility that resembled actual clinical conditions [28].

- Group 1: 15 degrees angulation.
- Group 2: 30 degrees angulation.
- Group 3: 60 degrees angulation.
- Group 4: 90 degrees angulation.

Three periodontally and systemically healthy volunteers were included in this study for the formation of in-vivo plaque biofilms on the four tested groups of implants. For four days (96 h), each volunteer wore a hard resin splint containing eight rough (machined implants; 3.5 mm in diameter and 8 mm in length) tightened with an orthodontic wire to allow dental plaque to naturally build up on the titanium surfaces of the implants [29].

**Fig. 1:**
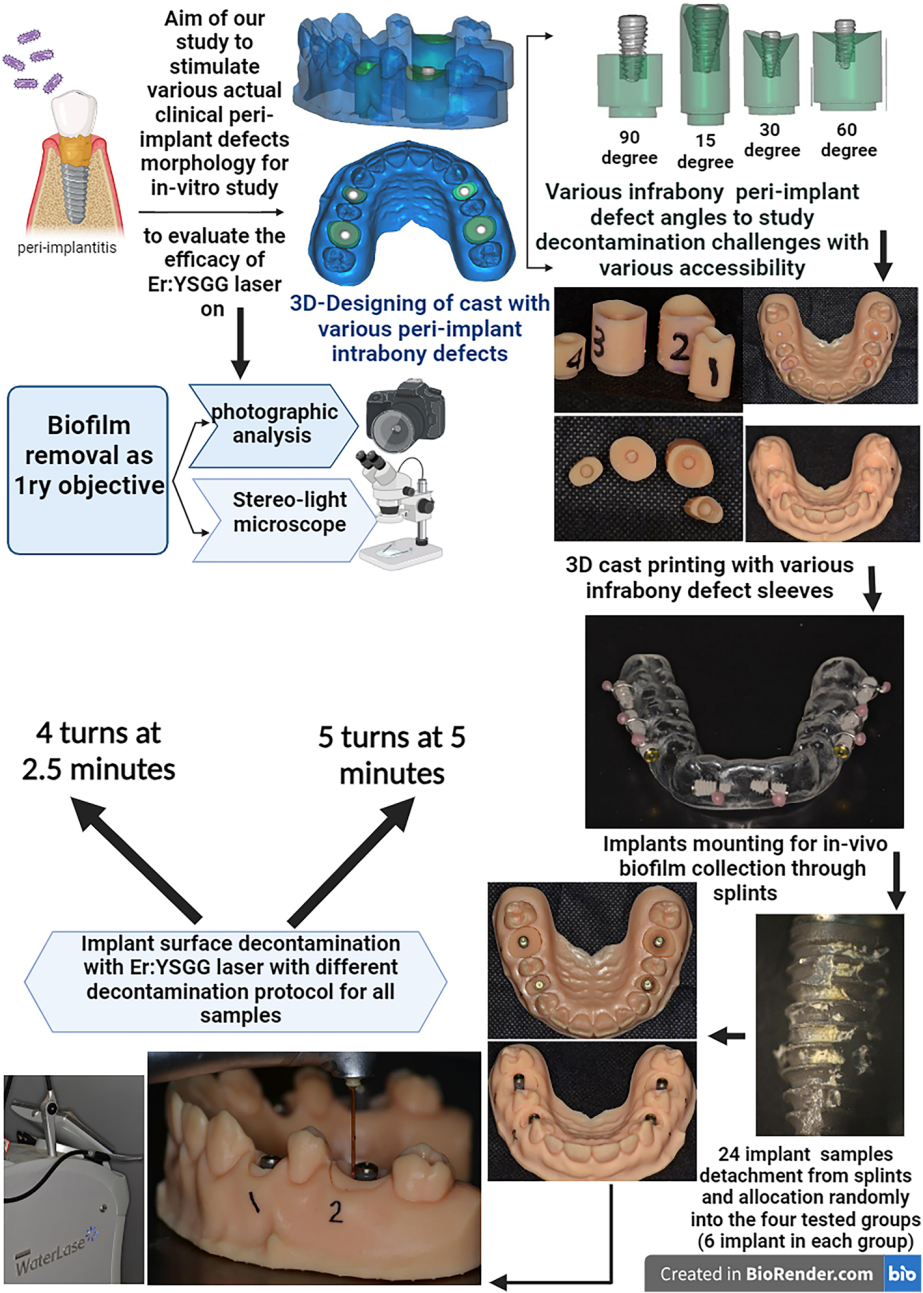
The flow chart of the study protocol

Afterwards, in various peri-implant bone defect simulators, the 24 implants that were collected after biofilm accumulation were assessed for Er,Cr: YSGG laser therapy efficacy in two protocols of application with the same laser device settings. These steps are summarized in (Fig. 1).

### Implant surface treatment

An Er, Cr: YSGG laser Waterlase Medical Device^1^ was used for decontamination of the samples. The laser beam was applied with a modified conical tip with a 500-micron diameter (0.5 mm) and 14 mm length (radial firing tip RFPT5-14) at the following parameters: 50 mJ (1.5 W/30 Hz), 50% air, 40% water in 0.5 mm distance non-contact mode in H-mode [30].

Two different decontamination methodology protocols were compared: 4 cycles at 2.5 min and 5 cycles at 5 min beginning from the most apical part of the defect going coronally in a circumferential motion and vice versa. The laser tip gradually moved along the implant surface within the coronal and apical ends in a circular motion and back again. This circular motion minimizes the neglected regions without decontamination [31, 32]. The difference between both protocols was the decontamination time and the number of decontamination cycles with identical laser beam settings. Both protocols were applied to four peri-implantitis simulating defects.

### Data collection

**Fig. 2:**
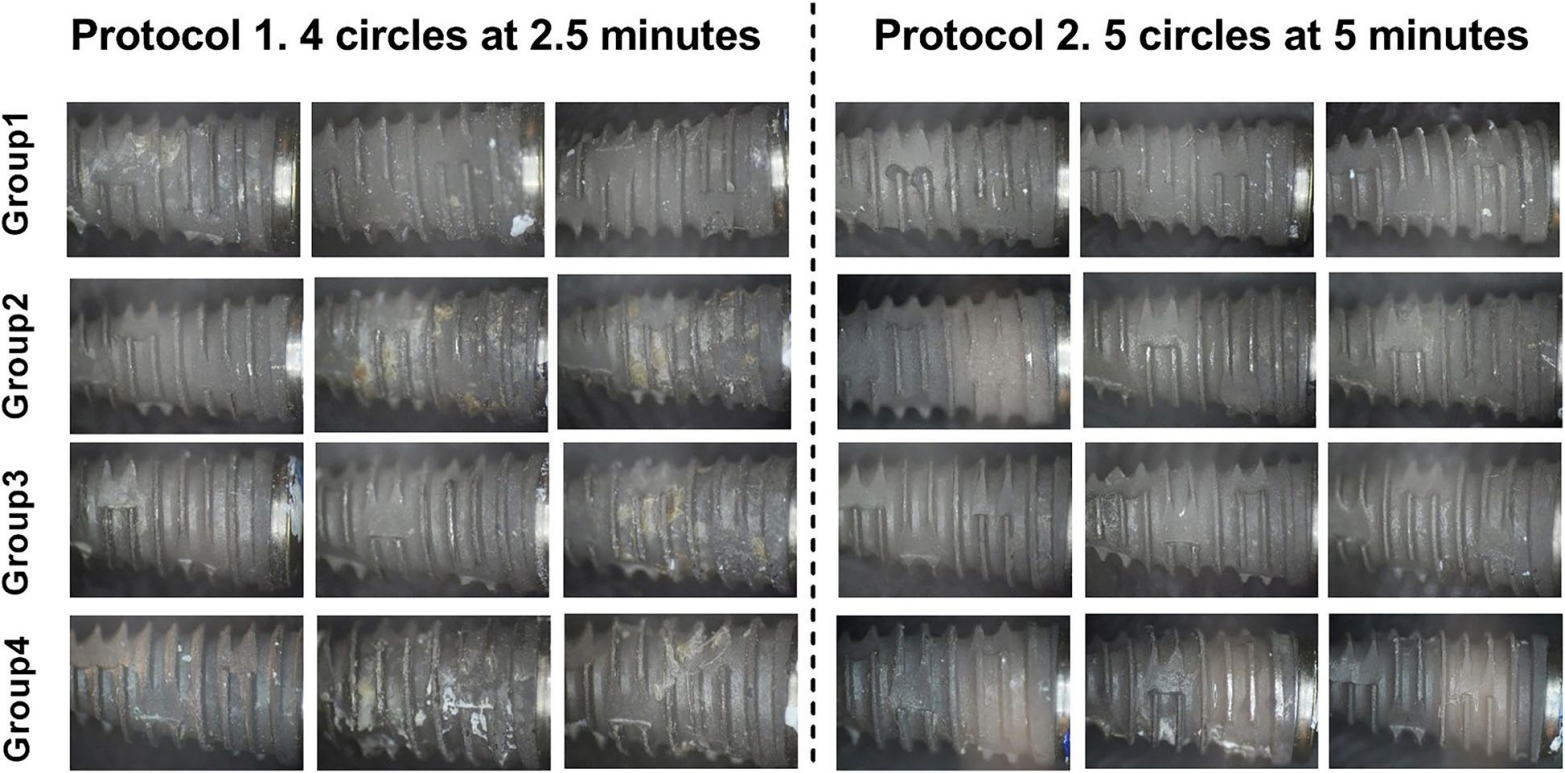
Photographic analysis of both protocols

Implant surface cleaning quality was determined in the study by measuring the biofilm removal capabilities using digital photos acquired from a digital video camera (Canon EOS 650D, Japan) under a stereo light microscope (LG-PS2, Olympus, Japan). Photomicrographs were taken at the original 2.5 × magnification without disclosing solution (Fig. 2).

For a total of twenty-four (24) implants, two pictures (2) were obtained from each side of each implant. Therefore, a collective blind analysis of 48 photos was performed for each protocol. Twelve photos from each group were analyzed separately in each protocol to determine the proportion of biofilm remnant on each surface for each group. After that, comparisons were made between different groups.

Digital plaque quantification of the photos was performed using image analysis software (Adobe Photoshop 2021, version 22.3.0 20210302.r.49). The following formula was used to obtain the biofilm percentage: area of remaining biofilm remnants × 100/total implant surface cropped area in line with other research designs [9].

### Statistical analysis

All collected data were statistically analyzed using one-way analysis of variances test (ANOVA), comparing the implant surface area (pixels) of the analyzed photos to reduce the bias regarding photo analysis, comparing the implant surface area/biofilm surface area ratio (%) in the four studied groups to detect the decontamination accuracy in various groups,

Also, a comparative analysis of the implant surface area/biofilm surface area ratio (%) in the two different laser application protocols (intragroup) using unpaired t-test and (intergroup) using Independent t-tests analysis were conducted.

## Results

**Fig. 3:**
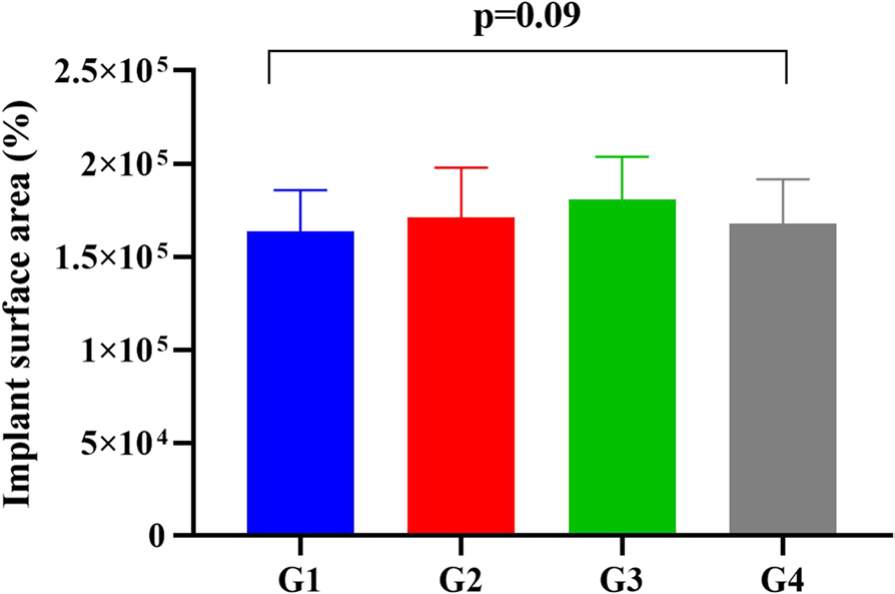
Bar chart illustrating the mean implant surface area(pixels) in the four studied groups

Initially, a comparative analysis of the implant surface area (pixels) of the analyzed photos and a multi-comparative analysis of different groups with various peri-implantitis defect angulations^2^ using one-way analysis of variances test (ANOVA) were conducted for the different study groups. There was no significant difference among the four groups (F-value:2.2, p-value of 0.09). The data are presented as the mean and standard deviation (Fig. 3).

**Fig. 4:**
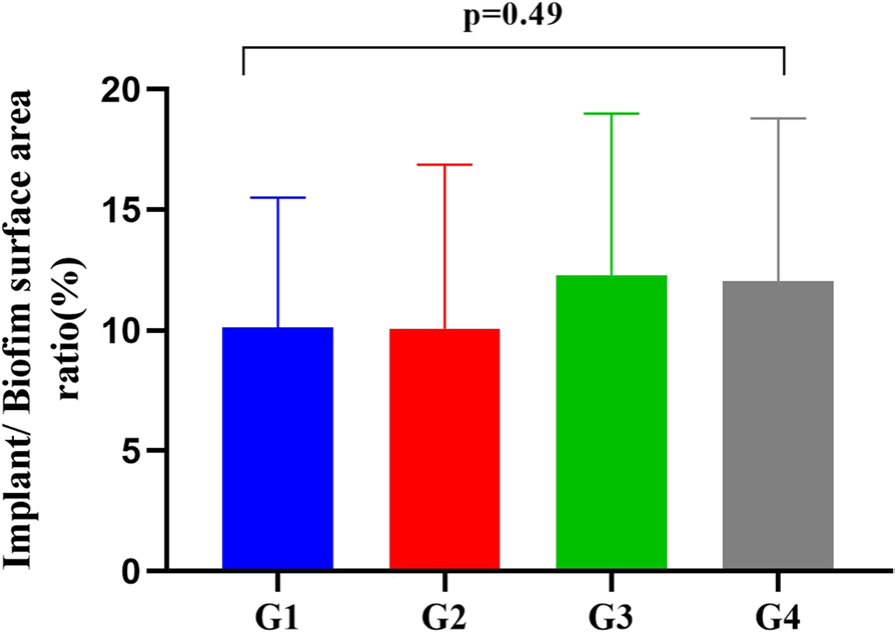
Bar chart showing no significant difference (*p* > 0.05) in the median values of the implant/biofilm surface area ratio (%) among the four studied groups

Then, a comparative analysis of the implant surface area/biofilm surface area ratio (%) in the four studied groups was conducted. There was no significant difference among the four groups (F = 0.81, *p* = 0.49).the data are presented as the mean and standard deviations. The results are presented in (Fig. 4).

Additionally, two comparative analyses of the implant surface area/biofilm surface area ratio (%) in the two different laser application protocols were conducted.^3^ One comparative analysis was performed in the same group (intragroup) and the other was performed between various tested groups (intergroup).

**Fig. 5:**
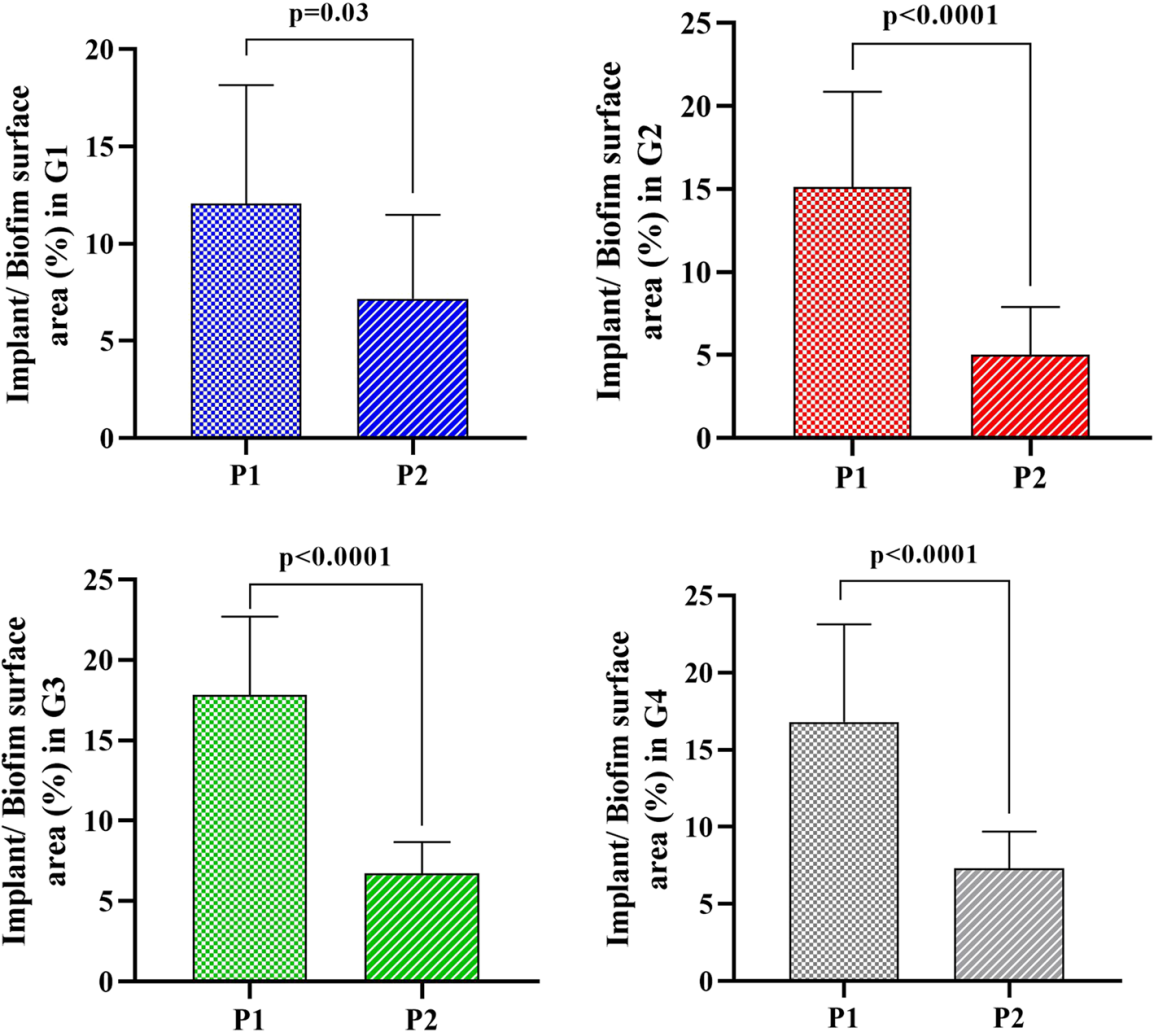
Bar chart showing a significant reduction in the mean implant/biofilm surface area ratio (%) obtained from protocol 2 (5 cycles at 5 min) in the four studied groups

First, comparative intragroup analysis was performed by comparing the implant surface area/biofilm surface area ratio (%) between the two different laser decontamination protocols in each studied group. The two applied protocols were compared in each group for the implant surface area/biofilm surface area ratio using the unpaired t-test. According to the obtained results, protocol 2 was significantly associated with a reduction in the % of implant surface area/biofilm surface area ratio in groups 2, 3, and 4 (*p* < 0.0001),; however, a mild reduction was observed in group 1 (*p* = 0.03). The results are presented in (Fig. 5).

**Fig. 6:**
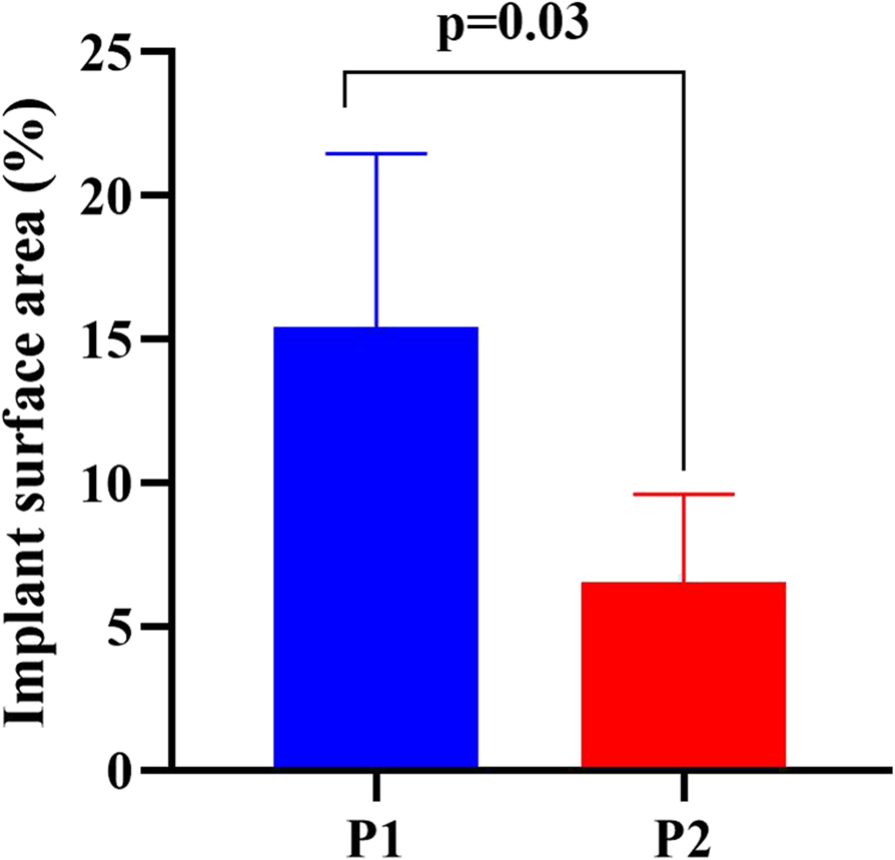
Bar chart showing a marked reduction in the mean implant/biofilm surface area ratio (%) obtained from protocol 2 (5 cycles at 5 min), compared to protocol 1 (4 cycles at 2.5 min) (*p* = 0.03)

Second, comparative intergroup analysis was performed by comparing the implant surface area/biofilm surface area ratio (%) between the two different laser decontamination protocols in various studied groups.^4^ Independent t-tests revealed that a marked reduction in the implant surface area/biofilm ratio (%) was significantly (*p* < 0.0001) associated with protocol 2 (mean: 6.56), compared to the mean value of (15.4) for protocol 1. The data are presented as the means and standard deviations. The results are presented in (Fig. 6).

## Discussion

In this era, laser therapies have been developed for peri-implant diseases treatment; therefore, they are becoming a growing focus of modern clinical applications and research with promising outcomes. However, there are still inconclusive clinical results, consequently, further well-controlled preclinical and clinical studies are essential to reach a precise conclusion regarding the ultimate parameters combined with the laser delivery system in peri-implantitis therapy since both influence the power density and spatial distribution of energy delivered to the implant surface. Wavelength modifications have implications for laser energy and the degree of absorption into the implant surface, biofilm, and adjacent target tissues. Additional variables such as tip design or tip diameter and shape, and how these terminal parts of the delivery system interact with gingival crevicular fluid, blood, or water. Maximum implant surface decontamination with full disinfection as well as the inactivation of toxins such as lipopolysaccharides without causing deleterious changes to the implant surface are essential for successful therapy [21].

The main hypothesis of this study was the Er, Cr: YSGG laser clinical efficacy evaluation as an adjunctive tool in peri-implantitis therapy. The purpose of this study was to determine the integral factors that influence the success of peri-implantitis therapy, as the efficiency of the decontamination process (biofilm removal) at various peri-implant defect was the primary objective.This study assumed that increasing the laser application time and number of cycles would enhance the decontamination efficacy.Therfore, the secondary objective was comparing between two protocols the first protocol (4 cycles at 2.5 min) and the second protocol (5 cycles at 5 min).

Regarding the attained results; The Er, Cr: YSGG laser was proved to be statistically significant in biofilm percentage reduction in various peri-implantitis defects.Therfore,this device with the reported settings is suitable decontamination tool in peri-implantitis therapy.This efficacy was claimed in the literature [30].

An incidence of missing areas with inadequate decontamination of implant surfaces was proposed, even with the same trained operator. The literature reported the impact of defect morphology, suprastructure, and operator experience. Thus, careful, proficient operator manipulation was crucial [10, 33, 34].

In this study, the decontamination processes in group-1 and group-4 were more challenging with a repetitive incidence of accidental contact with the implant surface or inner wall of the infrabony defect. However, group-2 and group-3 were more accessible during decontamination. This was obvious in intragroup comparative results, which showed a significant reduction in the % of implant surface area/biofilm surface area ratio in groups 2, 3, and 4,; however, a mild reduction was observed in group 1 (15 degrees of bone defect angulation). This confirmed that defect angulation influenced the width of the defect with suspected limited accessibility and decontamination efficacy. Therefore, defect flaring is supposed in narrow defect angulations clinically.

Unfortunately, no similar studies have compared to the obtained results. The other study that compared decontamination efficacy with the same defect angulations depended on other decontamination modalities used for peri-implantitis therapy [28].

Other studies with different modalities proved the influence of defect angulation and decontamination modality on the decontamination process. One study by Giffi supposed that the 60° bone defects were the easiest to debride, and the 30° defects were the most difficult that supported the results of this study [34, 35].

The significant reduction in the implant surface area/biofilm surface area ratio (%) in protocol 2 through comparative inter-group analysis was suspected to be a result of the time of application elevation in protocol 2 (5 cycles at 5 min) compared to protocol 1 (4 cycles at 2.5 min). These results confirmed the importance of determining the time of laser application in laser therapy.

This decontamination superiority of protocol 2 was parallel with other study postulated that increase the number of debridement applications (more passages number) was more efficient than a single passage [36].

Therapeutic results of any studied diseas, such as peri-implantitis,are influenced by study-type variables and restrictions. In the field of peri-implantitis therapy, for example, there is an overall scarcity of different in-vivo research, preclinical animal studies, and human clinical trials that impact the choice of the final therapeutic program.There are distinguishing issues concerning in-vivo studies than the clinical reality they could influence the accuracy of the results, such as the employed samples that differ from ordinarily threaded titanium implants (titanium discs, cylinders, strips, and sheets), biofilm complexity issues and structure (nonmineralized supragingival plaque, single-species biofilm, bacterial products, artificial ink), the scarcity of utilized custom-made defect models with various morphologies mimicking peri-implant defects in actual clinical settings simulation (specialized patient characteristics, such as peri-implant defect variations), saliva and blood influence on the accessibility of infected implant surfaces, the existence of the suprastructure, any other anatomical limitations such as tongue or soft tissues, and reinfection vulnerability [37].

Hence, the shortcomings of this research include the general drawbacks of ex-vivo and in-vivo studies regarding sample selection, biofilm utilization or other biofilm simulators, and defect model planning. To overcome the lack of comparable research and attain more clinical reality, we made an effort to mimic actual clinical settings.

Regarding the selected methodology strength and limitations; First, regarding the selected sample, similar to previous research, we decided to assess a commercially available screw-shaped implant with more clinical reality rather than alternative assessed samples such as discs and cylinders [29, 38, 39].

The discs and cylinders utilized in other studies did not resemble commercially available implants because of their complicated micro and macro topographic structures [40]. Discs and cylinders have comparable topographies across different sites that differ from the meticulous topography of screw-type commercially available implants [41]. In addition, the plaque growth pattern on titanium discs inserted in an intraoral splint system does not reflect the real clinical situation encountered at the transmucosal part of screw-type titanium implants [42].

The collection of failed explanted implants from peri-implantitis sites has also been suggested in some studies. However, because of the long period of sample collection, less sample availability is suggested, and various sample characteristics are a serious problem preventing constant comparability [43].

Second, regarding the simulation of various peri-implantitis defects allowing the assessment of Er, Cr: YSGG laser therapy with varying accessibility, this study was planned to generate four inconstant morphological experimentally created defects that allowed the simulation of several degrees of accessibility to the peri-implant defect, as naturally, the peri-implant lesions did not have an intimate configuration. The standard height and width of the defects with only angulation variance were used to minimize the variables in the study. However, the clinical situation is further complicated by countless varieties of observed peri-implantitis defects. Due to the variety of clinical depth, angulation, and width of the defects, the presence of abutments or soft tissue, as in nonsurgical therapy protocol, could influence decontamination efficacy [28]. Other similar studies have attempted to verify the correlation between decontamination efficacy and variably created defects [28, 39].

Third, regarding biofilm structure and complexity, as in similar studies, this study assessed invivo-developed biofilms. This is still the more realistic type to investigate [29]. This study was assumed to allow biofilm accumulation on implant surfaces for 4 days to allow more time for biofilm accumulation and maturation. Initial homogenous and mature biofilm was proven to cover the titanium discs in situ after 24 h, but after 4 days, biofilm accumulation was proven to be significantly thicker with greater biomass than with 1-day of accumulated biofilm [44].

In contrast to other in-vitro studies that utilized other unrealistic biofilm simulating protocols that did not represent real clinical situations, such as the use of different ink materials to simulate biofilms in laboratory-designed studies [9, 38, 39].

Furthermore, other studies have assessed in vitro-developed biofilms created under pre established conditions with limited bacterial species, limited complexity, and quantitative differences. In contrast, in-vivo established biofilms are created with more complex bacterial species in the presence of a broad range of nutrients and appropriate environments that represent more profitable colonization with more complex bacterial communities [44, 45].

Fourth, regarding laser therapy protocol, serious conflicts in laser parameter selection, techniques, and devices have been discussed in the literature, with unlimited varieties of various laser parameters without single advocated parameters. Therefore, the modification of some parameters in response to others is still accepted. Consequently, all the various parameters must be evaluated together [6]. In this study, Er: Cr: YSGG laser was used. It is an efficient tool without negative alterations or a temperature increase risk in a 0.5 mm noncontact mode with the following decontamination parameters: 50 mJ (1.5 W/30 Hz), 50% air, and 40% water, which is safe for implant surfaces [30].

The selected power in this study was 1.5 watts, which has been suggested for use in multiple studies [6, 30]*.* The parameter range restrictions for safety imclude avoiding undesirable surface alterations such as melting and flattening that are detected at higher powers of 2W and 3W [46]. Following the manufacturer’s guidelines, the H-mode (short pulse) was selected because this mode is preferred for hard-tissue therapy. However, the S-mode (long pulse) is recommended for soft tissue therapy. Therefore, H-mode was selected [47].

The explanation behind the selection of a side-firing tip in peri-implantitis therapy is the logistical achievements of delivering laser energy to the threads or subgingival regions of a dental implant effectively without noticeable implant surface alterations. This was a consequence of the decrease in the beam intensity in the frontal (distal) direction (by as much as 49%) due to the lateral emission of laser energy. Furthermore, this tip was evidently valuable for directing the laser beam laterally to the implant surface and soft tissue walls of the pockets, and enhancing accessibility with improved access to narrow bony defects and adjacent implant fixtures [21, 48].

The selected circumferential irradiation in both decontamination protocols compared in this study was established to stabilize the decontamination process in actual clinical infrabony defects, where visual control of biofilm removal is not applicable. In contrast to other studies, which have not reported clear decontamination methodologies or have depended on visual judgment until complete contaminant disappearance [49]. The selected time of application in the literature is vague or applied to other samples,such as titanium discs that are unapplicable for meticulous implant geometry. The reported time of application in the literature (120 s) has been denied to be sufficient for biofilm removal from the implant surface [50]. The applied focal distance of 0.5–1 mm during laser application that was selected in this study is intended for the avoidance of bringing the laser tip too close to the titanium surface of the implant with a higher power density [51].

## Conclusion

Both compared decontamination protocols of Er, Cr: YSGG are effective in reducing the total implant surface area/ biofilm ratio (%) at 50 mJ (1.5 W/30 Hz), 50% air, and 40% water, so it is suitable to utilize Er, Cr: YSGG with these settings as decontamination tool in peri-implantitis therapy. However, the second protocol strategy with an elevated time of application (5 cycles at 5 min) had a markedly significant reduction compared to the first protocol (4 cycles at 2.5 min). Therefore, the time of application and the defect depth are integral issues that influence the success of the decontamination process. Additionally, the angle of the defect influenced the decontamination accuracy, as only a mild reduction was observed in group 1(15 degrees of bone defect angulation) between both protocols.

### Supplementary Information

Supplementary Material 1

Supplementary Material 2

## Data Availability

All data generated or analyzed during this study is included in this published article and its supplementary information files.

## Abbreviations

Er,Cr: YSGG laser: (Erbium, Chromium: Yttrium, Scandium, Gallium, Garnet) laser
CAD: Computer‐aided design

## Footnotes

1 Waterlase MD (Biolase, Irvine, California, USA).

2 Group 1: 15 degree of bone defect angulation, Group 2: 30 degree of bone defect angulation, Group 3: 60 degree of bone defect angulation, and Group 4: 90 degree of bone defect angulation. ANOVA: Analysis of variances.

3 Protocol 1″ 4 cycles at 2.5 min”, and P2: protocol 2″ 5 cycles at 5 min.

4 Protocol 1″ 4 cycles at 2.5 min”, and P2: protocol 2″ 5 cycles at 5 min.
