# Supplementary material for: Comparison of the efficacy of Er,Cr:YSGG laser on oral biofilm removal from implant surfaces with various application times for the treatment of peri-implantitis defects: ex vivo study
Source: BMC Oral Health. 2024 Aug 22;24:980. doi: 10.1186/s12903-024-04698-5 (PMC11342501; doi:10.1186/s12903-024-04698-5)
Supplement: Supplementary file 1 — Supplementary Material 1 [file 12903_2024_4698_MOESM1_ESM.docx]

Additional

| **Group** | **first protocol 4 circles at 2.5 minutes** | | |
| --- | --- | --- | --- |
| Group 1 | **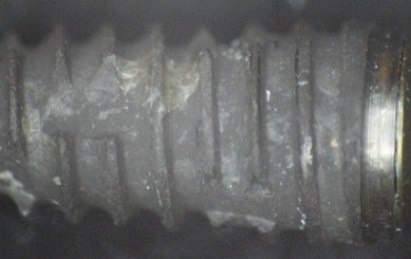** | **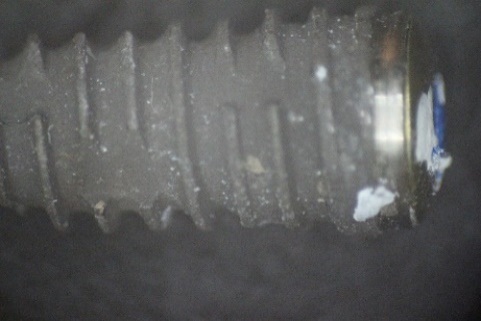** | **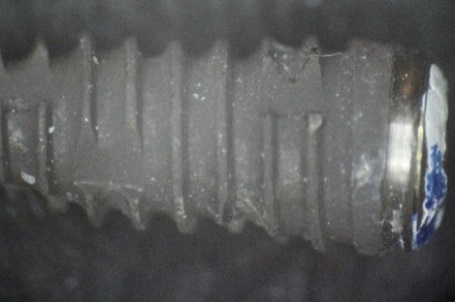** |
| Group 2 | 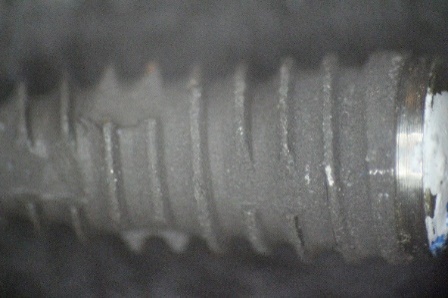 | **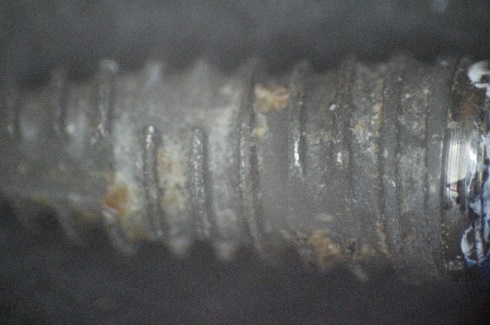** | **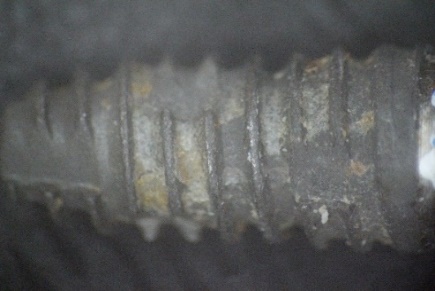** |
| Group 3 | **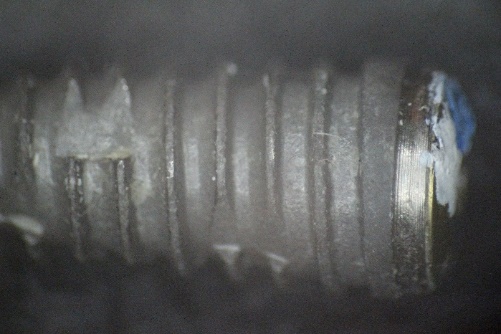** | **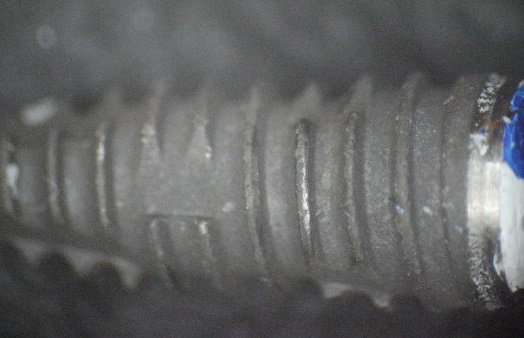** | 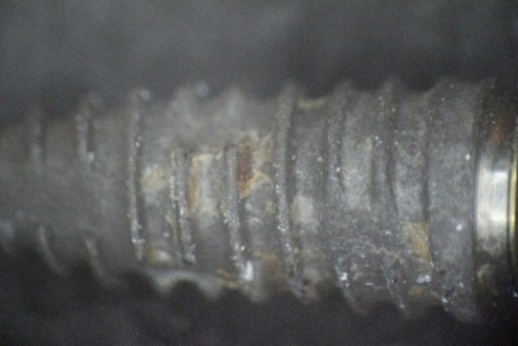 |
| Group 4 | **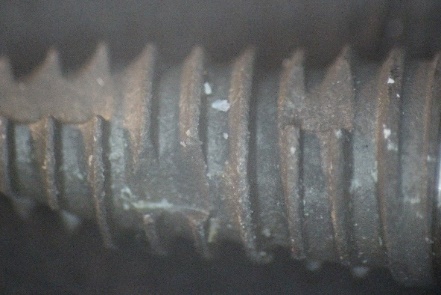** | **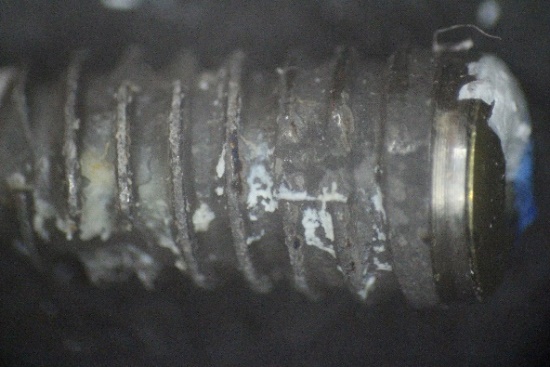** | **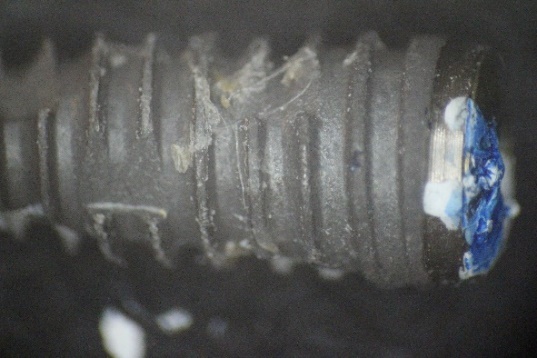** |
| **Group** | **Second protocol 5 circles at 5 minutes** | | |
| Group 1 | 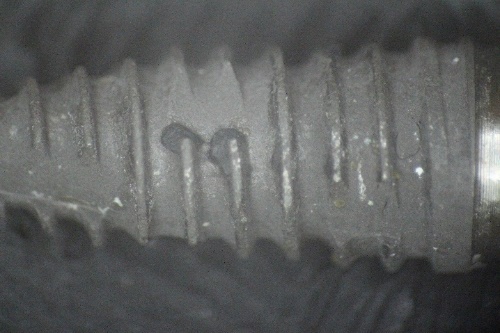 | 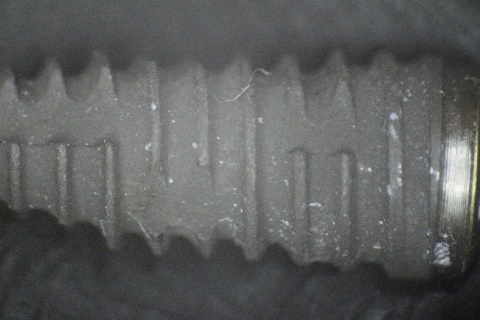 | **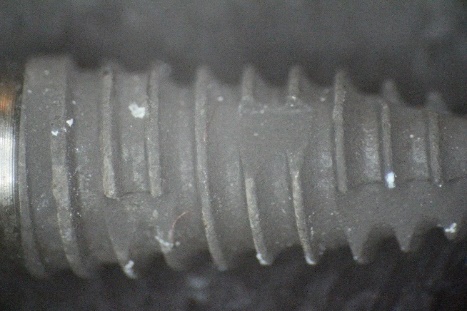** |
| Group 2 | 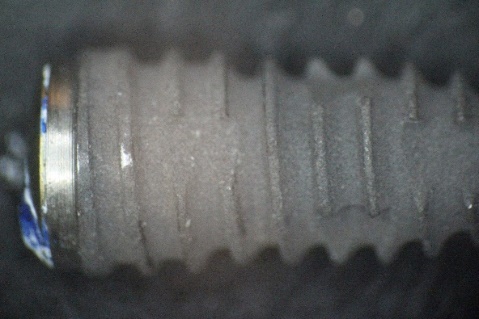 | **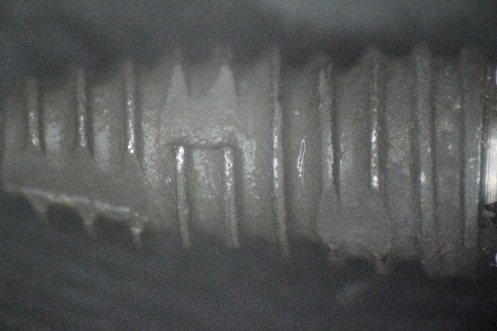** | **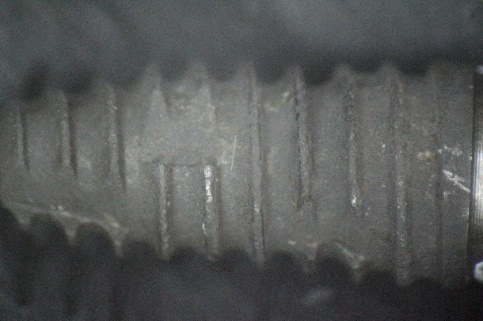** |
| Group 3 | **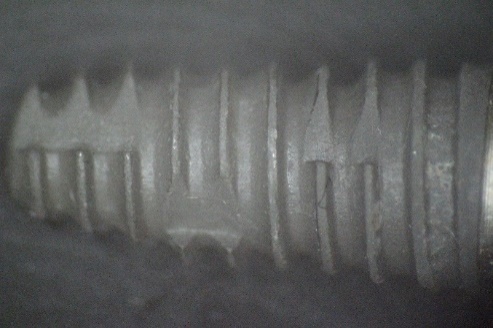** | **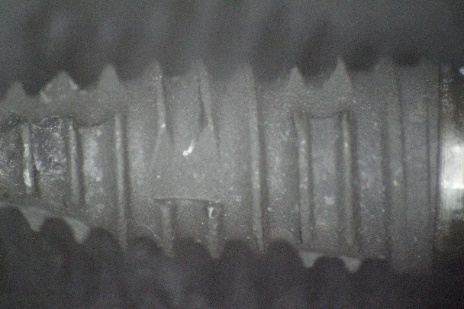** | 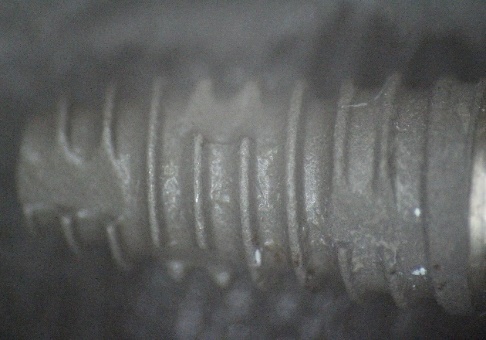 |
| Group 4 | **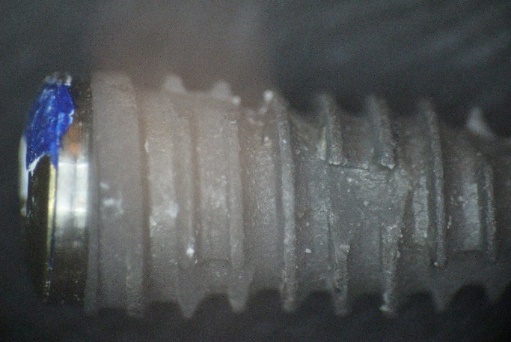** | **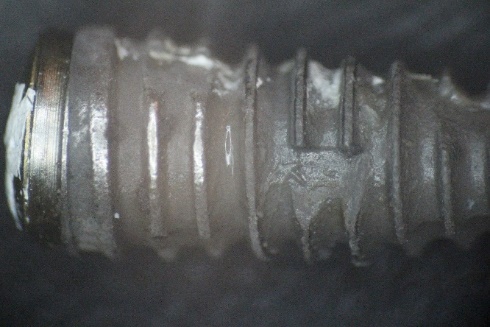** | **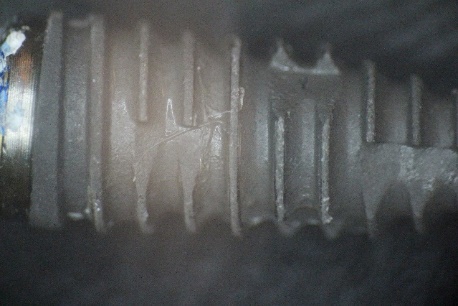** |

**Comparative analysis between implant surface area (pixels) in different studied groups:**

**Table 1: Comparative analysis between the implant surface area (pixels) of the examined groups (ANOVA) ^^[[1]](#footnote-1)^^.**

|  | **Group 1 (n=24)** | **Group 2 (n=24)** | **Group 3 (n=24)** | **Group 4 (n=24)** |
| --- | --- | --- | --- | --- |
| **Mean SD±** | 163464±22256 | 171119±26742 | 180614±23127 | 167586±24004 |
| **Range** | 132986 - 200812 | 131524 - 205070 | 139562 - 209950 | 124344 - 217946 |
| **Statistics** | F=2.213, p=0.09 [NS] | | | |

**Table 2: Multi-comparative analysis between different groups for the implant surface area (pixel) of the examined groups (ANOVA****)** ^^[[2]](#footnote-2)^^.

| **Tukey's multiple comparisons test** | **Mean Diff.** | **95.00% CI of diff.** | **Adjusted P Value** |
| --- | --- | --- | --- |
| G1 vs. G2 | -7655 | -25852 to 10542 | 0.6900 [NS] |
| G1 vs. G3 | -17150 | -35347 to 1047 | 0.0722 [NS] |
| G1 vs. G4 | -4122 | -22319 to 14075 | 0.9340 [NS] |
| G2 vs. G3 | -9495 | -27692 to 8702 | 0.5242 [NS] |
| G2 vs. G4 | 3533 | -14664 to 21730 | 0.9570 [NS] |
| G3 vs. G4 | 13028 | -5169 to 31225 | 0.2467 [NS] |

**Comparative analysis between the selected biofilm surface area (pixel) in different studied groups:**

**Table 3: Comparative analysis between the Biofilm surface area (pixel) of the examined groups comparative)** ^^[[3]](#footnote-3)^^.

|  | **Group 1 (n=24)** | **Group 2 (n=24)** | **Group 3 (n=24)** | **Group 4 (n=24)** |
| --- | --- | --- | --- | --- |
| **Mean ±SD** | 16544 ± 31753 | 4717 ± 34652 | 5611 ± 46169 | 7678 ± 51166 |
| **Range** | 3633 - 200812 | 131524 - 205070 | 139562 - 209950 | 124344 - 217946 |
| **Statistics** | F=1.45, p=0.042 [S] | | | |

**Table 4: multi-comparative analysis between different groups for the Biofilm surface area (pixel) of the examined groups (post-Hoc) test)** ^^[[4]](#footnote-4)^^.

| **Tukey's multiple comparisons test** | **Mean Diff.** | **95.00% CI of diff.** | **Adjusted P Value** |
| --- | --- | --- | --- |
| **G1 vs. G2** | -833.5 | -9434 to 7767 | 0.9942 [NS] |
| **G1 vs. G3** | -6178 | -14779 to 2423 | 0.2439 [NS] |
| **G1 vs. G4** | -3498 | -12098 to 5103 | 0.7121 [NS] |
| **G2 vs. G3** | -5345 | -13945 to 3256 | 0.3692 [NS] |
| **G2 vs. G4** | -2664 | -11265 to 5936 | 0.8493 [NS] |
| **G3 vs. G4** | 2680 | -5920 to 11281 | 0.8470 [NS] |

**Comparative analysis between implant surface area/biofilm surface area ratio (%) in four studied groups:**

**Table 5: Comparative analysis between the implant surface area/ biofilm ratio (%) of the examined groups (non-parametric)** ^^[[5]](#footnote-5)^^.

|  | **Group 1 (n=24)** | | **Group 2 (n=24)** | | **Group 3 (n=24)** | **Group 4 (n=24)** |
| --- | --- | --- | --- | --- | --- | --- |
| **Median**  **(25^th^-75^th^ percentile)** | | 10.1 (5.42 – 14.9) | | 7.72 (3.95 – 16.7) | 10.5 (7.37 – 17.0) | 10.9 (7.39 – 14.0) |
| **Range** | | 2.12 – 19.5 | | 3.30 – 23.8 | 10.5 (3.40 – 29.1) | 10.9 (4.6 – 14.0) |
| **Statistics** | | F=0.81, p=0.49 [NS] | | | | |

**Comparative analysis between implant surface area/biofilm surface area ratio (%) by two different protocols(inter-group).**

**Table 6: Comparative analysis between implant surface area/ biofilm ratio (%) obtained from the two approaches used for implants in the four studied groups (independent t-test)** ^^[[6]](#footnote-6)^^.

| **Group** | **P1(n=24)**  **mean±SD (range)** | **P2 (n=24)**  **mean±SD (range)** | **Statistics** |
| --- | --- | --- | --- |
| **G1 – G4 (n=96)** | 15.4±6.1 (0.12 – 29.1) | 6.56 ± 3.06 (2.12 – 18.8) | t= 9.11, p<0.0001[HS] |

**Comparative analysis between** **implant surface area/biofilm surface area ratio (%) by two different protocols in each studied group****(intra-group).**

**Table 7: Comparative analysis between implant surface area/ biofilm ratio (%) obtained from the two approaches used for implants in each group (independent t-****test)** ^^[[7]](#footnote-7)^^.

| **Group** | **P1(n=12) mean±SD (range)** | **P2 (n=12) mean±SD (range)** | **Statistics** |
| --- | --- | --- | --- |
| **Group 1 (n=24)** | 12.1±6.1 (0.12 – 19.5) | 7.2 ± 4.3 (0.12 –19.5) | t= 2.3, p<0.03[S] |
| **Group 2 (n=24)** | 15.1±5.8 (7.0 – 23.8) | 5.03 ± 2.9 (3.30 – 13.7) | t= 5.43, p<0.0001[HS] |
| **Group 1 (n=24)** | 17.8±4.88 (11.8 – 29.1) | 6.74 ± 1.95 (3.40 – 9.22) | t= 7.31, p<0.0001[HS] |
| **Group 2 (n=24)** | 16.8±6.35 (10.9 – 27.9) | 7.30 ± 2.39 (4.60 – 12.5) | t= 4.84, p<0.0001[HS] |

1. **^ANOVA:^** ^Analysis of variances, F: ANOVA test value, NS: no significant difference between the studied groups,^  [↑](#footnote-ref-1)
2. **^CI:^** ^confidence of interval,^ **^NS:^** ^no significant difference between the studied groups.^

   **^Group 1:^** ^15 degrees of bone defect angulation,^ **^Group 2:^** ^30 degrees of bone defect angulation,^ **^Group 3:^** ^60 degrees of bone defect angulation, and^ **^Group 4:^** ^90 degrees of bone defect angulation^. [↑](#footnote-ref-2)
3. **^ANOVA:^** ^Analysis of variances^**^, F:^** ^ANOVA test value^**^, S:^** ^significant difference between the studied groups^ **^(p<0.05),^** [↑](#footnote-ref-3)
4. **^CI:^** ^confidence of interval,^ **^NS:^** ^no significant difference between the studied groups.^

   **^Group 1:^** ^15 degrees of bone defect angulation,^ **^Group 2:^** ^30 degrees of bone defect angulation,^ **^Group 3:^** ^60 degrees of bone defect angulation, and^ **^Group 4:^** ^90 degrees of bone defect angulation^. [↑](#footnote-ref-4)
5. **^ANOVA:^** ^Analysis of variances^**^, F:^** ^ANOVA test value,^ **^NS:^** ^no significant difference between the studied groups (p>0.05),^

   **^Group 1:^** ^15 degrees of bone defect angulation,^ **^Group 2:^** ^30 degrees of bone defect angulation,^ **^Group 3:^** ^60 degrees of bone defect angulation, and^ **^Group 4:^** ^90 degrees of bone defect angulation^. [↑](#footnote-ref-5)
6. **t=** unpaired t-test value, **HS:** High significant difference between the studied groups (p<0.001), **G1** group 1; 15 degrees of bone defect angulation, **G2** Group 2: 30 degrees of bone defect angulation, **G3** Group 3: 60 degrees of bone defect angulation, **G4** Group 4: 90 degree of bone defect angulation, **P1:** protocol 1( 4 cycles at 25 minutes), **P2:** protocol 2( 5 cycles at 5 minutes). [↑](#footnote-ref-6)
7. **t=** unpaired t-test value, **S**: mild significant difference between the studied groups (p<0.05), **HS**: High significant difference between the studied groups (p<0.001), **G1** group 1; 15 degrees of bone defect angulation, **G2** Group 2: 30 degrees of bone defect angulation, **G3** Group 3: 60 degrees of bone defect angulation, **G4** Group 4: 90 degrees of bone defect angulation, **P1:** protocol 1( 4 cycles at 25 minutes), **P2:** protocol 2( 5 cycles at 5 minutes). [↑](#footnote-ref-7)
